# *Meloidogyne incognita* population control and nutritional status and productivity of Thompson seedless grapevines managed with different treatments

**DOI:** 10.1371/journal.pone.0239993

**Published:** 2020-10-06

**Authors:** Mahmoud Abdel-Sattar, Amr M. Haikal, Sandy E. Hammad

**Affiliations:** 1 Department of Plant Production, College of Food Science and Agriculture, King Saud University, Riyadh, Saudi Arabia; 2 Pomology Department, Faculty of Agriculture, Alexandria University, Alexandria, Egypt; 3 Horticulture Department, Faculty of Agriculture, Damanhour University, Damanhour, Egypt; 4 Department of Plant Nematology Research, Plant Pathology Research Institute, Agricultural Research Center, Giza, Egypt; University of Delhi, INDIA

## Abstract

A two-year field trial was conducted in a vineyard (northern Egypt)cultivated with Thompson seedless grapevines to evaluate the effectiveness of four “alternative” (biological/chemical) treatments, *Bacillus megaterium*, boric acid, calcium nitrate and chitosan, against the root-knot nematode *Meloidogyne incognita* (*Mi*), compared to that of the nematicide oxamyl. The influence of these treatments on plant nutritional status and fruit yield and quality was also assessed. All treatments significantly inhibited *Mi* reproduction parameters in both seasons, decreasing the numbers of nematode galls and egg masses (roots) and of second-stage juveniles (soil). Oxamyl application resulted in the highest reductions in *Mi*-reproduction parameters, followed by boric acid, which also showed the highest relative nematicidal efficacy (respect to oxamyl). In the 1st season, the highest fruit yield (10.34 kg/grapevine) was recorded from boric acid-treated plants, followed by that from oxamyl-treated plants (7.50 kg/grapevine); in the subsequent season (2019), oxamyl use led to the highest yield, followed by boric acid + chitosan use (10.04 and 8.62 kg/grapevine, respectively). In both seasons, application of boric acid alone and combined with chitosan enhanced the total soluble solids (TSS)/total acidity ratio in grape juice. All treatments led to higher nutrient contents (leaf petioles) and chlorophyll levels (leaves) as well as enhanced fruit size and weight. We conclude that the tested treatments can be safely applied for nematode management in Thompson seedless grapevines, with positive effects on fruit yield and quality.

## 1. Introduction

Grape (*Vitis vinifera* L.) is one of the most important commercial fruit crops in the world and the first major deciduous fruit crop in Egypt, ranking in the second position after citrus. The total grape-planted area has increased in the last years in Egypt, especially in newly reclaimed lands, covering about 827373ha. Most of this area is already fruitful, accounting for a total production of 1,686,706 tons, based on the latest Agriculture Statistics published by the Egyptian Ministry of Agriculture [1]. The seedless variety “Thompson” is very popular in Egypt both for fresh consumption as a table grape and also to produce raisins for exportation [2].

Plant-parasitic nematodes (PPNs) impose a big challenge in most cultivated areas worldwide. It has been estimated that these widespread soil inhabitants cause a yield loss of about 12.3% annually, equivalent to approximately $157 billion dollars [3]. PPNs have expanded widely in the Egyptian vineyard soils [4]. Their feeding habit on grapevine roots impairs water and nutrients uptake as well as root and shoot growth, resulting in reduced vine vigor and yield losses [5, 6]. PPNs can also increase vine’s susceptibility to other pathogens, including viruses [7, 8]. Although there are many PPN species attacking grapevines throughout the world, not all species cause significant economic damage [9].

The root-knot nematodes (RKNs), *Meloidogyne* spp., are highly destructive PPNs in grapevines [6, 10]. Various methods for RKNs management are used in vineyards; chemical control is one of them [6]. Despite their high nematicidal potency, it has severe negative effects on the environment [11]. Therefore, there is an urgent need to find safer and less harmful alternatives to the use of synthetic nematicides control RKNs [5]. Some commercial bioproducts containing microorganisms as an active ingredient are currently available in the market [12]. The rhizobacteria *Bacillus megaterium* has shown nematicidal activity against *Meloidogyn*e spp., as well as a positive effect on various crops, which was associated to growth promotion [13]. The commercial product Bio-arc^TM^ is a biological nematicide containing *B*. *megaterium*; its positive action on the nutritional status of grapevines, which could, in turn, result in increased weight and number of clusters, and consequently, in higher yields, has been documented [10, 14].

Since the effect of PPNs is more harmful to plants that grow in nutrient-deficient soils as compared to those cultivated in richer soils, soil nutrition is a relevant factor that can be manipulated to adversely influence nematodes and benefit plants [15]. In this sense, Carneiro *et al*. [16] reported that RKNs attacks to plants are usually accompanied by mineral deficiencies. Thus, a balanced supply of macro and micronutrients is essential for plants to withstand nematode injury and increase resistance or tolerance to this pathogen infection [17].

Boron is a plant micronutrient essential for healthy growth and normal development of reproductive tissues [18–20]. In Mediterranean-type soils, both boron deficiency and boron toxicity are field-scale problems related to the narrow concentrations range that separate these two conditions [21]. It has been reported that boron excess can be toxic to plants and affect their growth, while its deficiency results in low and poor crop quality [18]. This micronutrient plays a pivotal role in early flowering [22], and its deficiency can limit pollen germination and normal pollen tube growth, affecting fruit set in grapes [23]. Under field conditions, it has been found that boron has significant nematicidal activity against the root-knot nematode *Meloidogyne incognita*, infesting Thompson seedless grapevines [5], and against the citrus nematode *Tylenchulus semipenetrans*, infesting orange trees [24]. These reports inform that boron application reduced nematodes’ final populations and also improved grape and orange fruit yield and quality. According to Couto *et al*. [25], boron application controlled *M*. *incognita* population. Another important plant nutrient is calcium, which is considered a structural component of plant cell walls [26] and the development and structural integrity of plant membranes [27]. It was reported that the shortage of calcium in the soil affects plant structures and make plants less resistant to nematode infections [28].

Another promising substance for the control of PPNs is chitosan, a naturally-occurring compound obtained from seafood shells [29]; this compound was found to be effective for the management of various diseases in many crops [30]. At an adequate dose, chitosan effectively controlled root-knot populations and soybean cyst nematodes [31–34]. Chitosan provides good nematicidal activity without affecting beneficial soil microbes [35], thus becoming an environment-friendly biofertilizer that can be used to improve crop yield without risk of environmental contamination, as it does not generate pesticide residues [29].

Oxamyl is one of chemical nematicides in Egypt, which was shown to be useful to minimize the impact of PPNs and increase grape [10]. Although chemical nematicides are more effective in reducing nematode-associated yield losses than biological-based methods, in many regions, there are ongoing research efforts to find more environment-friendly strategies to manage nematode infections in economically relevant crops [5, 36].

The main objective of this research was to identify alternatives strategies for the effective management of root nematodes in grapevines other than the use of synthetic nematicides. To this purpose, we analyzed the effects of some alternative treatments (including simple minerals, biofertilizers, and bioagents) not only on RKN population but also on the productivity of Thompson seedless grapevines, aiming at producing healthier and less polluted grapes, free of chemical residues, to meet the exportation requirements, with the additional advantage of lower costs.

## 2. Materials and methods

### 2.1. Site description and vineyard management

The present study was conducted during two successive production seasons (2017/2018 and 2018/2019) in a12-year-old private vineyard located in El-Sahrawi village, El-Beheira Governorate, Egypt. The selected experimental plots included Thompson seedless grapevines growing on sandy soil; they were uniform in growth and vigor and had been planted at a distance of 2.5 × 1.75m in a soil naturally infested with *M*. *incognita*. The physical and chemical properties of the experimental soil (0–90 cm depth) were determined and are shown in Table 1.

**Table 1:** Physical and chemical properties of the experimental vineyard soil.

Physical properties				Chemical properties										
Soil fraction (%)			CaCO_3_ (%)	pH	EC (dS/cm)	Cations (mEq/L)				Anions (mEq/L)			C.E.C (mEq/100 g)	OM (%)
Sand	Silt	Clay				Na^+^	K^+^	Ca^2+^	Mg^2+^	Cl^–^	HCO_3_^–^	SO_4_^2–^		
92.5	4.2	3.3	2.65	7.85	1.95	12.5	0.15	3.5	2.85	11.5	3.2	4.3	2.96	0.28

Grapevines were irrigated with a drip-irrigation system consisting of two lines and four drippers per vine (8 L/h). The grapevines were trained on a three-wire trellis and pruned to 7 canes with 12 eyes along with 7 renewal spurs; in total, 98 buds per vine were left. Standard agricultural practices for commercial grape orchards were carried out.

### 2.2. Experimental treatments

Group 1 (Ct): untreated, positive control. Vines continued growing on the soil naturally infested with *M*. *incognita* and received no treatment.

Group 2: standard chemical treatment. The nematicide oxamyl (Vydate 24% SL), produced by DuPont company, was applied at the recommended dose (5mL/grapevine). This was the comparative treatment.

Group 3: biological treatment. The commercially available biological nematicide Bio-arc^™^ (Organic Biotechnology Company, Egypt), which contains25 × 10^6^ cfu/g of *Bacillus megaterium*, was applied at a dose of 10g/grapevine.

Group 4: boric acid (H_3_BO_3_) alone. This chemical compound was applied at a dose of 25g/grapevine.

Group 5: boric acid (H_3_BO_3_) combined with calcium nitrate [Ca(NO_3_)_2_]. The dose applied: was 12.5 g boric acid + 10 g calcium nitrate/grapevine.

Group 6: boric acid (H_3_BO_3_) combined with chitosan. The dose applied was12.5 g boric acid + 10 g chitosan/grapevine.

A total of 60 vines, as uniform as possible in growth and vigor, were selected and subjected to these treatments, with 5 replicates per treatment and 2 vines per replicate (the number of the treated vines for each applied treatment were 10 vines) (i.e., 6 treatments × 5 replicates × 2 vines per replicate = 60 vines). The experimental treatments were arranged in a randomized complete block design (RCBD), according to Gomez and Gomez [37]. In both tested seasons, each treatment was applied twice, on January 15 and on February15. The treatments were distributed under the canopy of each vine near the stem with 25 cm from each side and then irrigated. Grapevines received the standard agricultural practices according to the recommendation of Ministry of Agriculture, Egypt.

### 2.3. Nematode infection assessment

In both seasons, before the application of the treatments, soil samples were collected with an auger that made a hole of 10 cm in diameter giving a total of about 1000 cm^3^ soil from 20 and 30 cm depths from each side (about 25 cm away from the vine trunk). The collected subsamples from each vine were mixed in one compound sample from which a sample was examined to estimate the number of *M*. *incognita* second-stage juveniles (J_2_) per kg of soil using sieving and centrifugal flotation technique [38]; this data was considered the initial nematode population (P_i_). In addition, approximately 10 g root samples were collected from each site of the vine to determine the number of root galls (G) and nematode egg masses (EM) using an aqueous solution of phloxin B (0.15 g/L) for 15 minutes to clarify the nematode egg masses [39]. The same parameters were estimated after treatment applications using soil and root samples collected at the end of each season to represent the final nematode population (P_f_) and final numbers of nematode galls and egg masses (with consideration that the final population of the 1st season was deemed the initial population of the 2nd season). Species of *Meloidogyne incognita* was identified based on the perineal pattern of the adult female that dissected from the infected galled roots of grapevine [40]. The perineal pattern characteristics were described according to [41].

The nematode reproduction factor (Rf) was calculated for each treatment by dividing P_f_/P_i_ [42]. For each nematode population parameter (G, EM, and J2), the percentage of reduction(R) was calculated based on Mulla’s formula [43], as follows:

Reduction%(R)=100−[(C1/T1)×(T2/C2)×100];

where: C1 = pre-treatment value in the untreated control (Ct); C2 = post-treatment value in the untreated control (Ct); T1 = pre-treatment value in a given treatment; T2 = post-treatment value in that treatment. To compare the effectiveness of the four alternative treatments to that of the nematicide oxamyl 24%, the relative nematicidal efficacy (RNE) of each one was calculated based on nematode reproduction factors (Rf) and expressed as a percentage, using the following equation:

RNE=[1−(treatmentRf–nematicideRf/treatmentRf)]×100.

### 2.4. Grapevine nutrient status and total chlorophyll contents

Two weeks after the fruit set, leaf petioles were collected for mineral composition analysis. The leaves situated opposite to the grape clusters were used to this purpose, as suggested by Cottenie *et al*. [44]. Samples of 20 petioles per replicate were washed carefully with tap water and distilled water and subsequently dried at 70°C until constant weight. Dried petioles were ground using an electric mill and digested using sulfuric acid and hydrogen peroxide. Grapevine nutritional status was evaluated by the determination of leaf petiole mineral content [45]. Nitrogen (N) was estimated by the micro–Kjeldahl method [46]; phosphorus (P), by the method of Murphy and Riely [47]; and potassium (K), by flame photometry according to the method of Brown and Lilleland [48]. Calcium (Ca), magnesium (Mg), and iron (Fe) were determined using an atomic absorption spectrophotometer (Perkin Elmer 3300), according to Carter [49]. Boron (B) was colorimetrically determined using the carmine method, as described by Hatcher and Wilcox [50].

Total chlorophyll content (SPAD) was also determined two weeks after fruit set on10 leaves per vine, chosen among those situated from the 6^th^ or 7^th^ leaf to the tip of the growing branches, according to the method described by Yadava [51]. A Minolta Chlorophyll METER SPAD– 502 was used (Minolta camera, LTD Japan).

### 2.5. Grapevine reproductive performance and yield

The number of burst buds was determined one month after bud burst by simple counting, and the percentage of bud burst, bud fertility, and fruiting coefficient were calculated according to Bessis [52] and Gaser *et al*. [53], using the following equations:

budburst(%)=numberofburstbuds/totalnumberofbuds×100;

budfertility(%)=numberofclusters/totalnumberofbuds×100;and

fruitingcoefficient(%)=numberofclusters/totalnumberofburstbudspervine×100.

At harvesting time (middle July, both seasons), the fruit yield per vine was estimated by multiplying the number of clusters/vine × average cluster weight.

### 2.6. Fruit parameters of commercial relevance

A total of 30 clusters per treatment (3 clusters/vine) was used to obtain average values for the following physical characteristics: cluster’s weight (g), cluster’s length (cm), cluster’s width (cm), 100-berries weight (g), 100-berries volume (cm3), berry´s length (mm), berry’s width (mm). The shape index was calculated as the fruit length/fruit width ratio. Fruit chemical characteristics assessed in the berry juice were the percentage of total soluble solids (TSS) and acidity (TA). The first one was determined using a hand refractometer. Total acidity was measured according to the official methods published by the AOAC [54] and expressed as grams of tartaric acid/100mL of berry juice. The ratio between total soluble solids and total acidity (TSS/TA) was calculated [55].

### 2.7. Statistical analysis

Treatment effects were evaluated by performing one-way analysis of variance (ANOVA). Means were separated and compared using the least significant difference (LSD) test at 0.05 level of probability, according to Steel and Torrie [56]. The statistical analysis was carried out using the software SAS (Statistical Analysis System), version 9.13 [57].

## 3. Results

### 3.1. Nematode infection assessment

**Fig 1:**
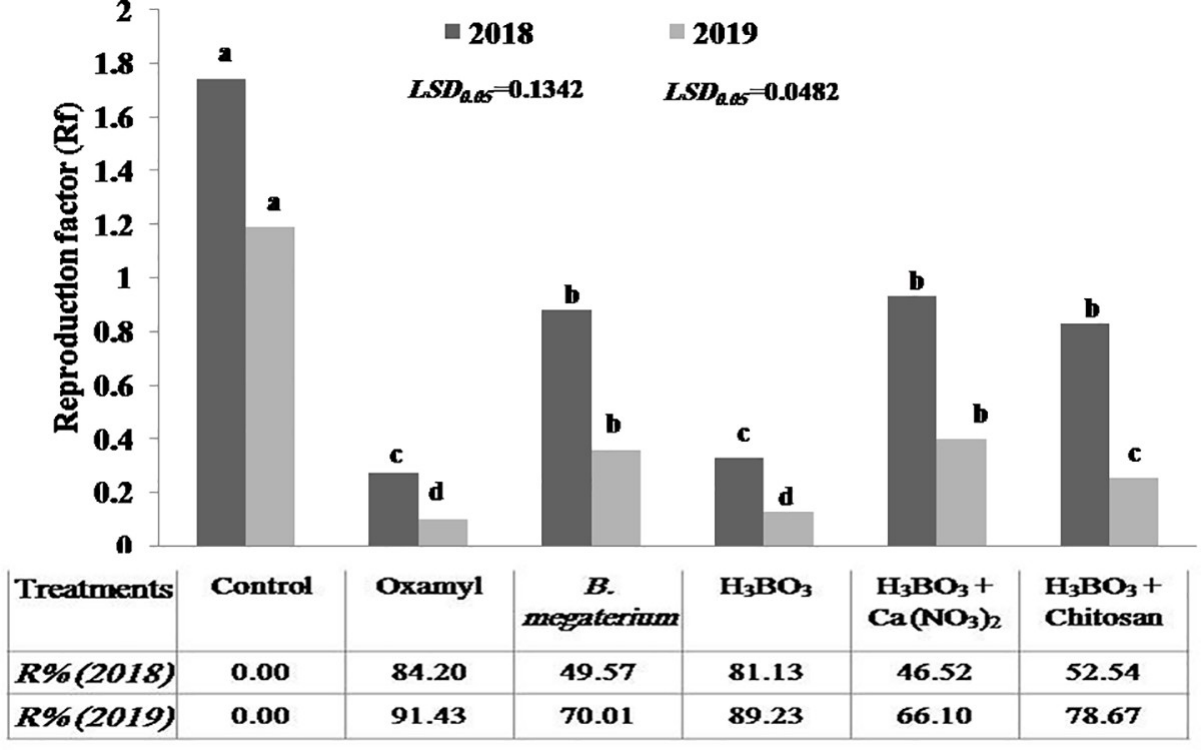
*Meloidogyne incognita* reproduction factor (Rf) in naturally infested vineyard soils subjected to different treatments. The reproduction factor (Rf) of *Meloidogyne incognita* and the reproduction factor reduction percentages (R %) were calculated as described in Material and Methods. Different letters indicate significant differences at P = 0.05. Data are means of 5 replicates.

Over both experimental seasons (2018 and 2019), all treatments significantly reduced the numbers of nematode galls and egg masses/g fresh root, as well as the numbers of second-stage juveniles (J_2_) per kg of soil and *M*. *incognita* reproduction factors (Rf) (Table 2 and Fig 1). It may be noticed that the application of boric acid alone was the most effective treatment, resulting in strong nematode populations decreases, without significant differences compared to the reductions observed with the use of the nematicide oxamyl 24%.

**Table 2:** *Meloidogyne incognita* density parameters in naturally infested vineyard soils subjected to different treatments.

**Treatment**	**Initial counts (2017)**			**Final counts 1st season (2018)**					
	**G**	**EM**	**J**_**2**_ **(P**_**i**_**)**	**G**	**R**	**EM**	**R**	**J**_**2**_ **(P**_**f**_**)**	**R**
**Mi(Ct)**	9.9	7.96	5381.4	12.44 a	-	11.12 a	-	9238.2 a	-
***B*. *megaterium***	9.94	7.36	5149.2	4.32 c	65.41	3.98 b	61.29	4519.2 bc	48.88
**H**_**3**_**BO**_**3**_	10.78	8.78	5653	2.62 d	80.66	2.48 c	79.78	1857.8 d	80.86
**H**_**3**_**BO**_**3**_**+ Ca(NO**_**3**_**)**_**2**_	9.9	7.04	5382.4	6.14 b	50.64	5.1 b	48.14	5018 b	45.69
**H**_**3**_**BO**_**3**_**+ chitosan**	11.16	8.12	4824	4.54 c	67.63	4.04 b	64.38	3998.8 c	51.71
**Oxamyl**	9.88	7.26	4976.2	2.24 d	81.96	1.74 c	82.84	1368.8 d	83.98
**L.S.D**_**0.05**_				1.4595		1.3939		612.26	
	**Initial counts (2018)**			**Final counts 2nd season (2019)**					
**Mi(Ct)**	12.44	11.12	9238.2	17.2 a	-	15.02 a	-	10944.6 a	-
***B*. *megaterium***	4.32	3.98	4519.2	1.80 c	69.86	1.46 bc	72.84	1606.8 b	69.99
**H**_**3**_**BO**_**3**_	2.62	2.48	1857.8	0.56 de	84.54	0.58 cd	82.69	238.2 d	89.18
**H**_**3**_**BO**_**3**_**+ Ca(NO**_**3**_**)**_**2**_	6.14	5.1	5018	2.94 b	65.37	2.56 b	62.84	2024.4 b	65.95
**H**_**3**_**BO**_**3**_**+ chitosan**	4.54	4.04	3998.8	1.64 cd	73.87	1.32 cd	75.81	987.6 c	79.15
**Oxamyl**	2.24	1.74	1368.8	0.30 e	90.31	0.22 d	90.64	141.6 d	91.27
**L.S.D**_**0.05**_				1.0861		1.2313		434.04	

Mi(Ct) = untreated, positive control. G = number of galls/g fresh root; EM = number of egg masses/g fresh root; P_i_ = nematode initial population of J_2_/kg soil; P_f_ = nematode final population of J_2_/ kg soil. *R* = reduction percentages, calculated using Mulla’s formula, as described in Material and Methods. Data are means of 5 replicates. Different letters within columns (for the same season) indicate significant differences at *P* = 0.05.

The reduction percentages in root-galls numbers (G) by applying the alternative treatments ranged from 50.64 to 80.66% and from 65.37 to 84.54% in 2018 and 2019, respectively. Vineyards treated with the nematicide oxamyl showed 81.96% and 90.31% reductions in this parameter in the 1st season (2018) and 2nd season, respectively. Similarly, the reduction percentages in nematode egg masses (EM) ranged from 48.14 to 79.78% (2018) and from 62.84 to 82.69% (2019) for the experimental treatments, whereas this parameter reached 82.84% in 2018 and 90.64% in 2019 with the use of the synthetic nematicide oxamyl. Table 2 also shows that the nematicide oxamyl 24% led to the highest reduction percentages in nematode juveniles (J_2_) final populations (P_f_) in soil, 83.98% (2018) and 91.27% (2019), followed by boric acid treatment, with 80.86 and 89.18% of reduction, while [boric acid+ calcium nitrate] treatment recorded the lowest reduction percentages: 45.69% (2018) and 65.95% (2019) (Table 2).

The reproduction factor (Rf) of *M*. *incognita* infecting Thompson seedless grapevine was significantly reduced by all treatments (Fig 1). Rf values ranged from 0.27 to 0.93 in the 1st season and from 0.1 to 0.4 in the subsequent season (2019) in treated soils, whereas in the untreated soils, Rf values were 1.74 and 1.19 (2018 and 2019, respectively). The lowest Rf values were achieved by oxamyl treatment, and these values were not significantly different from those recorded for boric acid treatment. The reduction percentages (R%) of *M*. *incognita* Rf observed under the alternative treatments ranged from 46.52 to 81.13% in 2018, and reached higher levels, 66.1 to 89.23%, in 2019. Under Oxamyl treatment, these percentages were 84.2% (2018) and 91.43% (2019) (Fig 1).

**Fig 2:**
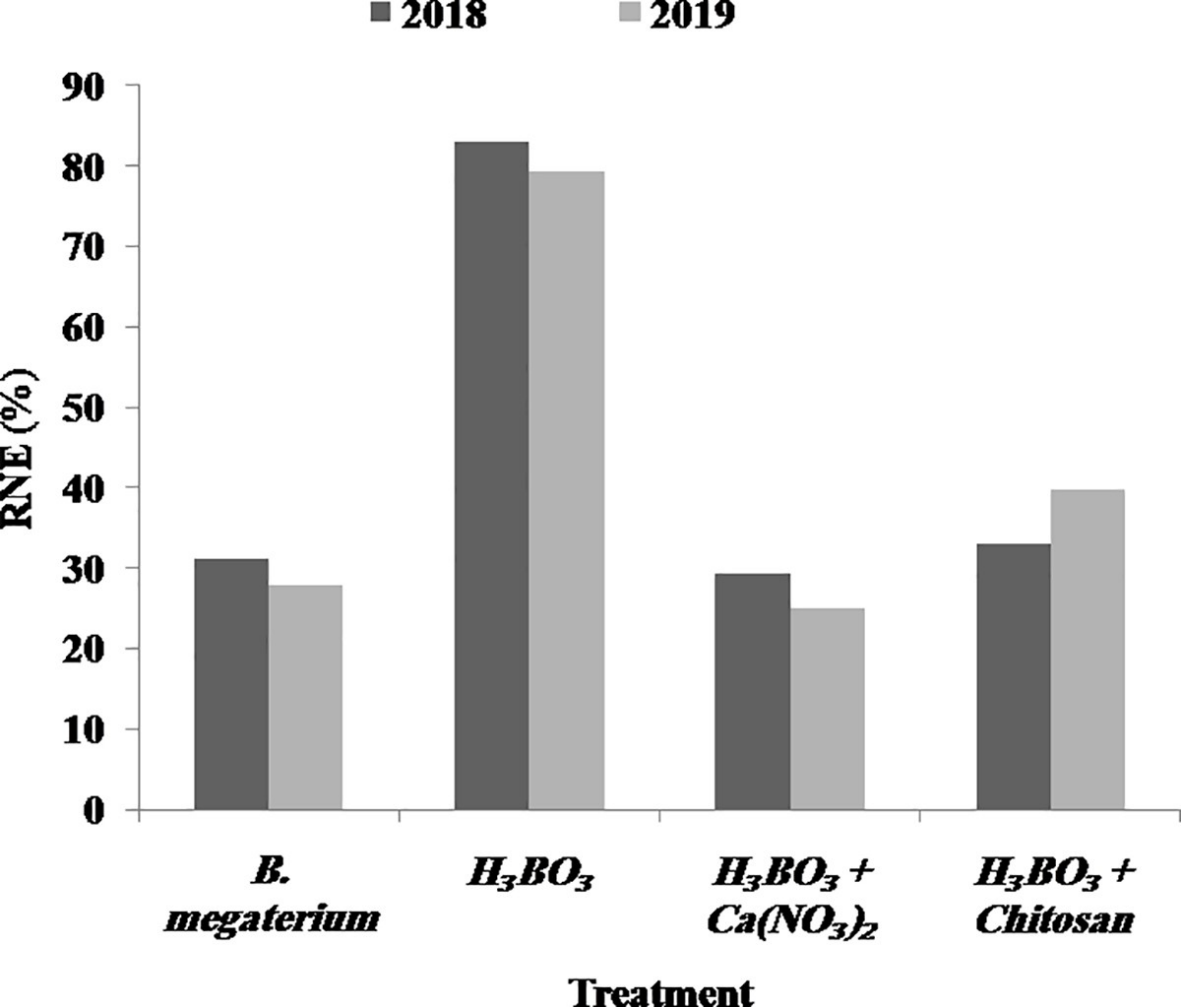
Relative nematicidal efficacy of different treatments. The relative nematicidal efficacy (RNE%) expresses the efficacy of each treatment against *Meloidogyne incognita*, compared to that of the nematicide oxamyl 24%, and was calculated as described in Material and Methods. Data are mean values of 5 replicates.

The relative nematicidal efficacy (RNE) of the alternative treatments respect to that of the nematicide oxamyl 24% is shown in Fig 2. It can be noticed that boric acid (H_3_BO_3_) applied alone behaved as the most potent agent against *M*. *incognita*, showing RNE of 83.03%in the 1st season and 79.37%in the 2nd season. However, the application of boric acid combined with other compounds resulted in lower RNE: 33.09% and 39.68% for [boric acid + chitosan] treatment, and 29.40% and 25% for [boric acid + calcium nitrate] treatment, in 2018 and 2019, respectively. The use of the bioproduct based on *B*. *megaterium* resulted in similar RNE compared to the combined treatments: 31.14% and 27.93% (1st and 2nd season, respectively).

### 3.2. Grapevine nutrient and chlorophyll contents

The concentration of several nutrients in the leaf petioles of Thompson seedless grapevine plants and total chlorophyll levels are shown in Table 3. In the 1st season (2018), the application of boric acid alone caused the most significant nitrogen (N), phosphorus (P), potassium (K), calcium (Ca), magnesium (Mg), iron (Fe), boron (B), and total chlorophyll (SPAD) rises. In the 2nd season (2019), however, [boric acid + chitosan] was the treatment responsible for the higher increments in leaf total chlorophyll and petiole nutrients except for boron, whose maximum concentration (89.8ppm) was recorded under boric acid application. In both seasons (2018 and 2019), all the other treatments caused significant increases in petiole nutrient levels and total leaf chlorophyll as compared with untreated vines (Ct) except for Oxamyl in the 1st season, which did not significantly elevate petiole potassium concentration (Table 3).

**Table 3:** Nutrient concentrations in leaf petioles and leaf total chlorophyll in Thompson seedless grapevines subjected to different treatments for controlling *Meloidogyne incognita*.

Treatment	1st season (2018)							
	N	P	K	Ca	Mg	Fe	B	Total chlorophyll (SPAD)
	(%)					(ppm)		
**Mi(Ct)**	1.76 e	0.15 f	1.09 d	2.08 f	0.40 e	122.6 f	20.8 f	24.05 e
***B*. *megaterium***	1.98 cd	0.20 d	1.20 c	2.34 d	0.53 d	145.2 d	27.0 d	27.42 cd
**H**_**3**_**BO**_**3**_	2.36 a	0.29 a	1.44 a	2.66 a	0.78 a	187.0 a	39.6 a	36.86 a
**H**_**3**_**BO**_**3**_**+ Ca(NO**_**3**_**)**_**2**_	2.03 c	0.21 c	1.24 c	2.40 c	0.58 c	153.6 c	33.6 c	28.19 c
**H**_**3**_**BO**_**3**_ **+ chitosan**	2.15 b	0.27 b	1.32 b	2.55 b	0.67 b	173.6 b	35.6 b	31.83 b
**Oxamyl**	1.92 d	0.19 e	1.12 d	2.27 e	0.5 d	141.4 e	25.0 e	26.90 d
**L.S.D**_**0.05**_	0.0561	0.0109	0.0542	0.0431	0.0391	3.7074	1.3162	1.1242
	**2nd season (2019)**							
**Mi(Ct)**	1.67 e	0.15 f	0.95 e	2.04 e	0.39 e	117.0 e	19.2 e	23.49 e
***B*. *megaterium***	2.17 c	0.22 c	1.40 b	2.54 bc	0.72 b	168.2 b	32.8 c	34.38 b
**H**_**3**_**BO**_**3**_	1.96 d	0.18 e	1.16 d	2.31 d	0.56 d	143.2 d	89.8 a	27.52 d
**H**_**3**_**BO**_**3**_**+ Ca(NO**_**3**_**)**_**2**_	2.24 b	0.26 b	1.43 b	2.55 b	0.73 ab	168.4 b	34.2 c	34.42 b
**H**_**3**_**BO**_**3**_ **+ chitosan**	2.40 a	0.27 a	1.49 a	2.82 a	0.76 a	176.0 a	39.0 b	36.71 a
**Oxamyl**	1.99 d	0.20 d	1.24 c	2.48 c	0.63 c	153.0 c	30.2 d	31.77 c
**L.S.D**_**0.05**_	0.0617	0.0109	0.0528	0.0696	0.0233	3.1974	2.8845	1.0898

Mi(Ct) = untreated control. Different letters within columns (for the same season) indicate significant differences at *P* = 0.05.

### 3.3. Grapevine reproductive performance and yield

**Fig 3:**
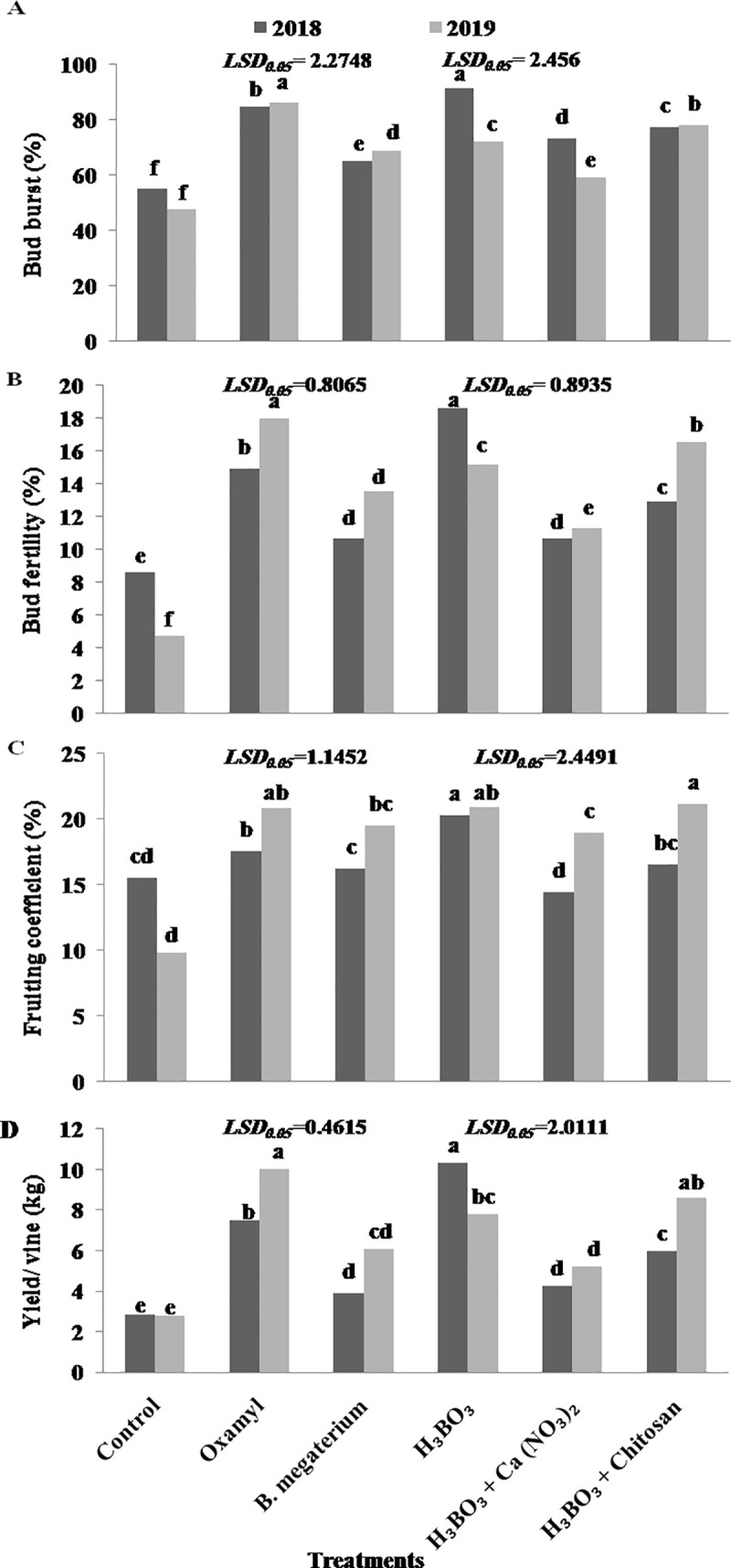
Effect of different treatments on the reproductive performance and yield of Thompson seedless grapevines. (A) Bud burst (%), (B) Bud fertility (%), (C) Fruiting coefficient (%), and (D) Yield (kg/vine). Different letters indicate significant differences at P = 0.05. Data shown are mean values of 5 replicates.

As a general trend, the applied treatments increased bud burst, bud fertility, and fruiting coefficient respect to the untreated control (Fig 3A, 3B and 3C). In the 1st season (2018), the exceptions were *B*. *megaterium*, [boric acid +calcium nitrate], and [boric acid + chitosan] treatments, in which the fruiting coefficients did not differ significantly from that observed in control grapevines. Bud burst, bud fertility, and fruiting coefficient reached the highest values in grapevines treated with boric acid: 91.43, 18.57, and 20.31%, respectively. In the 2nd season (2019), all treatments significantly improved the bud burst and bud fertility compared with the untreated control, and the highest bud burst and bud fertility percentages were recorded in oxamyl-treated vines, followed by vines under [boric acid + chitosan] treatment (Fig 3A and 3B). In terms of fruiting coefficient, however, [boric acid + chitosan] treatment was found to be slightly superior to boric acid and oxamyl treatments, being all of them over the control (Fig 3C).

Regarding grape yield, in the 1st season (2018), the highest value was obtained in grapevines treated with boric acid alone (10.34 kg/vine) followed by those which received oxamyl (7.50 kg/vine) (Fig 3D). In the subsequent season (2019), the highest yield was achieved in oxamyl-treated plants (10.04 kg/vine), followed by those under [boric acid + chitosan] treatment (8.62 kg/vine) and boric acid treatment (7.79 kg/vine). In both seasons, the lowest yields were recorded in the untreated control (Fig 3D).

### 3.4. Fruit parameters of commercial relevance

**Fig 4:**
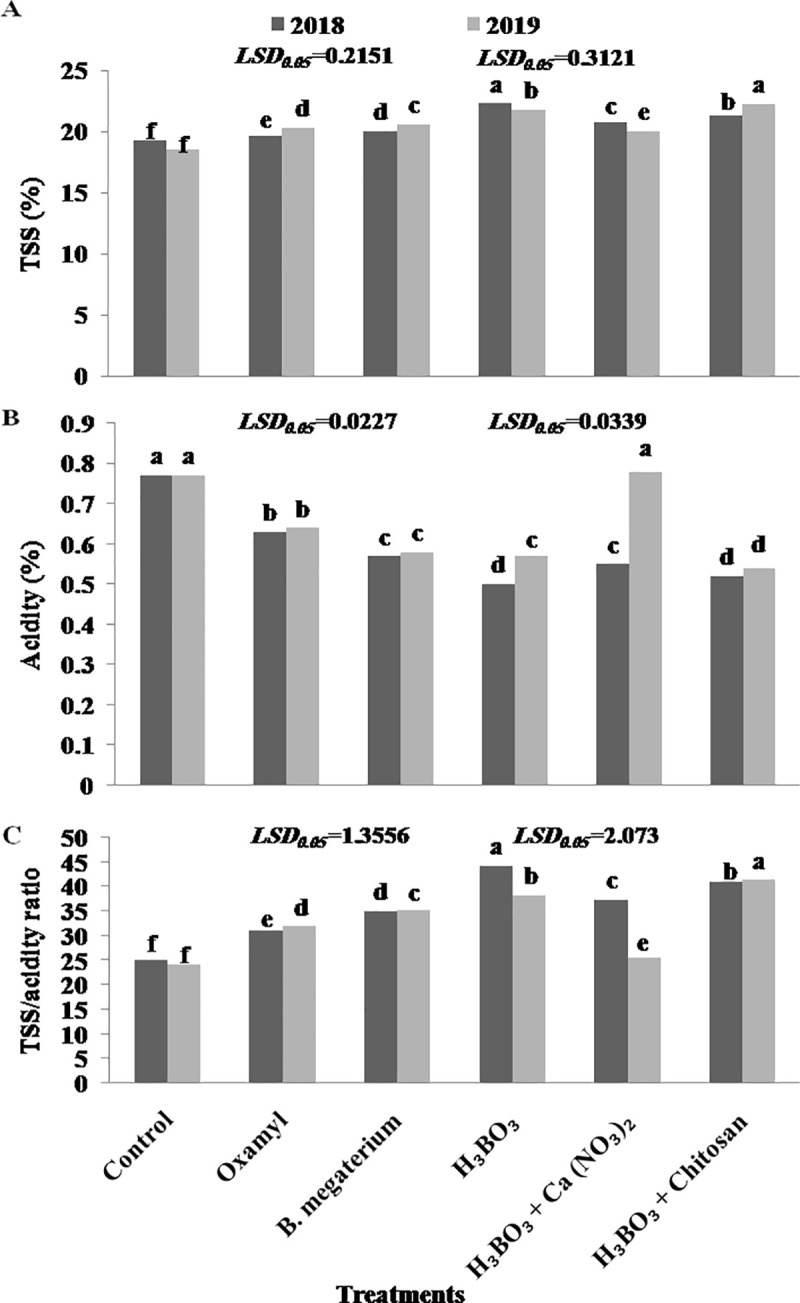
Effect of different treatments on chemical characteristics of the berry juice from Thompson seedless grapes. (A) TSS (%), (B) Acidity (%), and (C) TSS/acidity ratio. Different letters indicate significant differences at P = 0.05. Data shown are mean values of 5 replicates.

The juice of Thompson seedless grapevines harvested during 2018 and 2019 seasons was analyzed. In the 1st season (2018), the highest percentage of total soluble solids (TSS) was recorded in the grape juice obtained from boric acid-treated vines, followed by that obtained from [boric acid + chitosan] treatment (Fig 4A).

In the 2nd season (2019), conversely, the highest percentage of TSS was detected in [boric acid + chitosan]-treated vines, followed by that measured in the juice from boric acid-treated vines. In addition, both treatments (boric acid and [boric acid + chitosan]) significantly decreased the total acidity (TA) in both seasons (Fig 4B), resulting in significantly higher TSS/acid ratios (Fig 4C). Treatments with [boric acid + calcium nitrate], *B*. *megaterium*, and oxamyl had similar effects on these parameters (less pronounced but still significant compared to untreated plants) in 2018, while in 2019, [boric acid + calcium nitrate] addition led to a significant increase in TA, which resulted in a significant decrease in TSS/acid ratio. The highest TSS/TA ratios corresponded to boric acid-treated vines in 2018 and to [boric acid + chitosan] in 2019.

**Fig 5:**
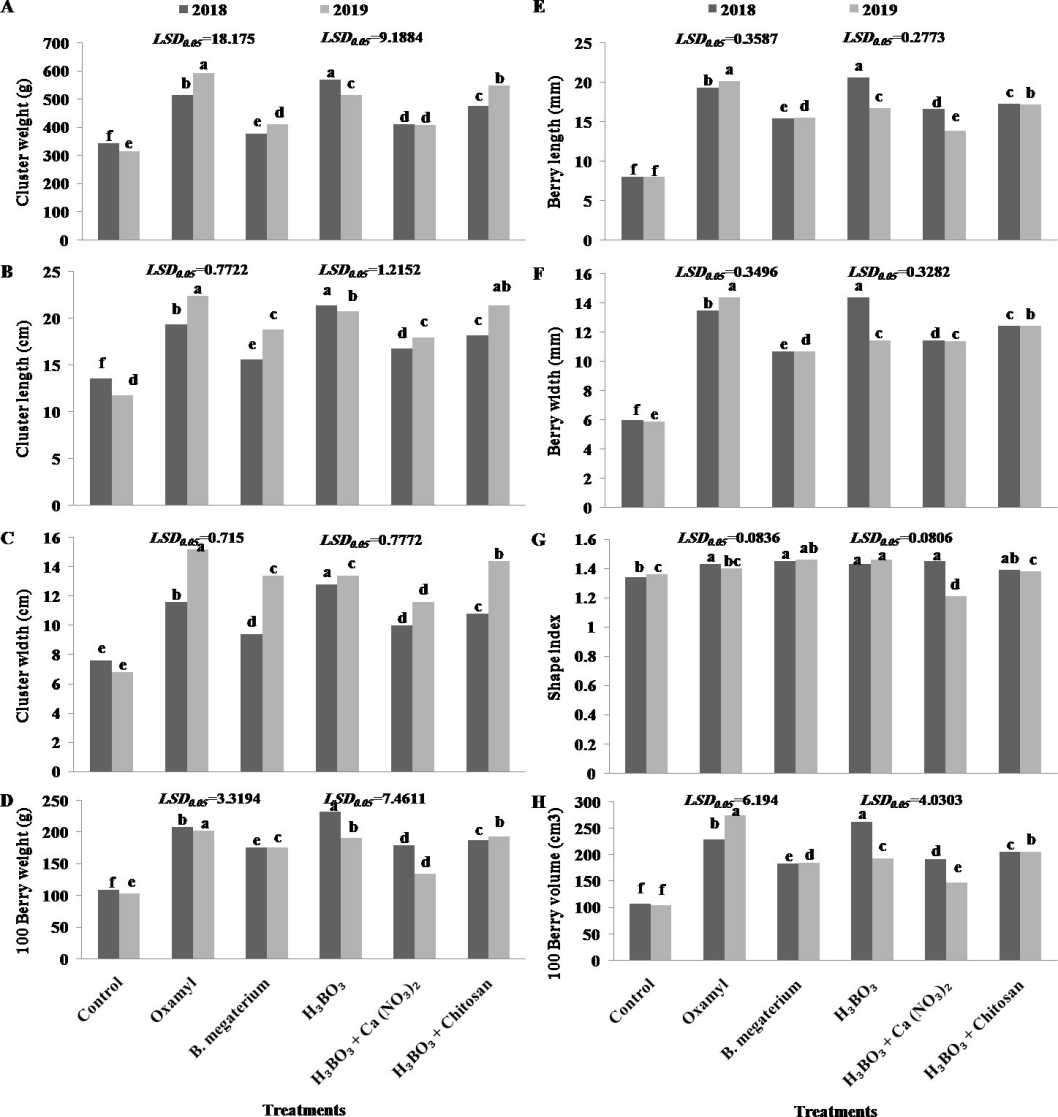
Effect of different treatments on relevant fruit parameters in Thompson seedless grapes. (A) Cluster weight (g), (B) Cluster length (cm), (C) Cluster width (cm), (D) 100 Berry weight (g), (E) Berry length (mm), (F) Berry width (mm), (G) Shape index, and (H) 100 Berry volume (cm3). Different letters indicate significant differences at P = 0.05. Data shown are mean values of 5 replicates.

Fig 5 shows the influence of different *M*. *incognita*-control treatments on relevant commercial characteristics of the harvested Thompson seedless grapevines. As it may be noticed, in both seasons the treatments applied improved these characteristics including cluster weight (Fig 5A), cluster length (Fig 5B), cluster width (Fig 5C), 100-berry weight (Fig 5D), 100-berry volume (Fig 5H), berry length (Fig 5E), and berry width (Fig 5F). In the 1st season, grapevines treated with boric acid displayed the highest values for these indices, followed by those treated with oxamyl. In the 2nd season, the highest values corresponded to grapevines treated with oxamyl, followed by those subjected to [boric acid + chitosan] treatment. Compared with the shape index (fruit length/fruit diameter width) of the untreated control, all treatments slightly increased this value in the grapes harvested in 2018 (Fig 5G). In those harvested in 2019, boric acid and *B*. *megaterium* treatments significantly increased this ratio, while [boric acid + calcium nitrate] treatment significantly reduced it. Treatment with [boric acid + calcium nitrate] generated the lowest increments respect to the untreated control in all physical characteristics assessed, except for the berry width, for which the lowest increment was associated with *B*. *megaterium* application (Fig 5F).

## 4. Discussion

Based on the assumption that mineral nutrition affects plants’ susceptibility to-parasitic nematode diseases, supplying plants with micronutrients may increase plant resistance against nematode pathogens. In this sense, Santana-Gomes *et al*. [17] stated that balanced plant nutrition could increase plant resistance or tolerance to nematode infection. Plants take up boron through the roots as uncharged boric acid (H_3_BO_3_) [58]. Although boron is a micronutrient, it was reported that its deficiency affects grapevine growth and fruit set and quality [59] because this element displays a role in fruit sugar accumulation and contributes to pollen germination, pollen tube growth, and normal fruit set [23]. However, boron excess can be toxic to plants affecting their growth [60]. In addition, fruit set clusters tend to be small under boron limitations, and berries cannot fully develop on the rachis [18].

Our results confirm the nematicidal potential of boric acid against the root-knot nematode, and they are in line with those reported by El-Saedy *et al*. [5], who showed that boric acid application significantly reduced the number of root galls and egg masses of *M*. *incognita* on the root system of Thompson seedless grapevines, along with improvements in fruit yield and properties of commercial importance. They also reported the significant nematicidal activity of boron against the citrus nematode, *T*. *semipenetrans*, infesting orange trees [24]. El-Saedy *et al*. [24] informed that boron treatment reduced the final nematode population and had a relative nematicidal efficacy (RNE) of about 83–85%. The application of this micronutrient also improved fruit yield and quality, compared to the untreated control.

While the nematicidal activity of boric acid against plant-parasitic nematodes (PPNs) has not been discussed in the literature, in 2006, Habes *et al*. [61] elucidated the mechanism involved in the toxicity of this compound to the nervous system of insects. These authors found that boric acid acts as an inhibitor of acetylcholine esterase (AChE) in the German cockroach, *Blatella germanica*. Since this mode of action coincides with that of many PPNs-targeting commercial nematicides [62], boric acid probably inhibited nematode AChE and thus reduced the nematode population. Surprisingly, boric acid was found to play a dual role in the present study, both as nematicide and fertilizer, and be as effective as oxamyl in controlling *M*. *incognita*.

Regarding grapevine reproductive performance and yield, our results show that high values were achieved in both seasons when boric acid, alone or in combination with chitosan, was applied. Besides, these treatments led to the highest percentages of total soluble solids (TSS) and the lowest percentages of total acidity (TA), resulting in significantly increased TSS/total acidity ratios. These results are consistent with those of previous reports, which revealed that boron application to vineyards increased grape yield [21, 63].

The improvement in grapevine growth following boric acid application may be attributed to nematode population control and probably to boron’s involvement in natural phytohormone transport, which may have contributed to both cell division and enlargement [5, 64]. Boron has a more critical role in the reproductive period than in the vegetative period [63]. Therefore, in soils with nutritive deficiencies or when plants cannot take up nutrients at the required levels, balanced fertilization is necessary to reach high grape yield [63]. Besides, it was reported that boron addition induces carbohydrates movement and ascorbic acid transfer from plant leaves to the fruiting bodies [21, 65], and this effect possibly led to the increase in the fruit weight in our research. Boron’s relevance in carbohydrate metabolism [66], cell division, protein synthesis [60], early flower initiation, and flower bud formation [22] was previously documented.

*M*. *incognita*-control treatments applied in this study had significant effects on leaf petioles nutrient contents in both tested seasons; they generally increased N, P, Ca, and Mg concentrations. Optimum grapevine growth has been associated with leaf nitrogen and phosphorus concentrations of 1.6–2.8% and 0.2–0.6%, respectively [67]; therefore, we consider that these nutritional requirements were met under the treatments tested in this study. Moreover, calcium and magnesium leaf contents were even above the optimum values proposed (0.4–2.5% and 0.13–0.4%, respectively) [67]. However, leaf potassium concentrations were below the optimum ranges reported by these authors (1.5–5.0%) [67].

The highest nutrient contents (leaf petioles) and total chlorophyll levels (leaves) were recorded for grapevines under boric acid and [boric acid + chitosan] treatments in the 1st and 2nd seasons, respectively. These results are in line with those reported by Gunes *et al*. [68], who mentioned that both foliar and soil applications of boron increased grapevine yield and the content of N, Ca, Mg, P, K, and Zn in both plant leaves and berry tissues.

Many studies document the nematicidal effect of chitosan against plant-parasitic nematodes, which is considered to depend on the concentration and molecular weight of this polymeric substance [30, 32, 33, 35]. It has been found that chitosan addition reduced root-knot nematode invasion by affecting parasite population parameters (i.e., number of galls, egg masses, females/root system, number of juveniles/250 g soil) and reducing *Meloidogyne* spp. egg hatching and larval viability [32, 34], for which this compound may serve as a natural nematicide. Moreover, chitosan applications were used in various fruits and vegetables to induce plant immunity, thus protecting them from different pests and pathogens [24]. Sharif *et al*. [29] reported that chitosan had fungicidal, bactericidal, and nematicidal activity.

The exact mode of action of chitosan to control pathogenic nematodes remains unclear, but it was proposed that chitin (the precursor of chitosan) may promote the growth of beneficial chitinolytic microbes that parasitize nematodes eggs [69]. Also, Sharif *et al*. [29] reported that chitosan treatment regulates the expression of several plant genes involved in plant molecular defense systems such as phytoalexins and pathogenesis-related proteins (PR)).

Our results are in line with those obtained by many researchers, who confirmed the positive effects of chitosan on the growth parameters of several crop plants. In grapevine, chitosan-treated plants showed better growth [70, 71]. Górnik *et al*. [72] found that chitosan behaved as a successful grapevine biostimulant, as its application improved the root system development and increased the number of internodes and newly formed canes, as well as their length; also enhanced leaf chlorophyll content.

Likewise, many researchers found that using chitosan as a coating material for fertilizers can control the release rate of the inorganic added nutrients, resulting in the prevention of excessive fertilization and lower production costs. It was also communicated that this practice improves the efficiency of fertilizers’ uptake by plants [73, 74]. Therefore, chitosan can be used as a biodegradable biofertilizer to avoid the hazards of inorganic fertilizers overuse in horticulture. Chitosan was also found to improve crop yield, and the shelf life of the harvested products, with less environmental contamination [29].

Calcium ion is considered one of the most important plant nutrients affecting plant susceptibility to diseases [75]. According to Hurchanik *et al*. [28], plants subjected to calcium deficiency are more susceptible to nematodes attack; therefore, soil calcium availability is a key point. Calcium content in plant tissues was related to disease incidence [17]. Pathogens growth and their chances to cause infection increase at higher sugar levels flowing from the cytoplast to the apoplast, a process that occurs mainly at low calcium levels. Besides, calcium ions can significantly inhibit the activity of the extracellular pectolytic enzymes (e.g., galacturonase) produced by various plant pathogens, thus preventing the middle lamella degradation and contributing to cell wall stability [75, 76]. It has been found that an increased calcium supply to plants promotes root cell resistance and thus reduces nematodes invasions to roots [75, 76].

Calcium can be applied to crops through different mineral fertilizers. For instance, calcium carbide addition reduced the number of galls, egg masses, and juveniles of *M*. *incognita* in zucchini, and it led to crop yield increases, which were positively correlated to the application rates [77]. Calcium sulfate application also resulted in a high reduction of *M*. *incognita* population density and improved tomato vegetative parameters [76].

Patil *et al*. [78] measured Ca(NO_3_)_2_ uptake by rice roots infected with the root-knot nematode *Meloidogyne graminicola* and found that soil nitrogen concentration and predominant form can influence the extent of root invasion by *M*. *graminicola* and, consequently, rice yield. These researchers showed that regulating soil nitrate concentration during *M*. *graminicola* infection periods can represent a non-chemical method of nematode management in rice crops.

Although grapevine growth requires microelements in small amounts, these nutrients may significantly affect grape properties [26]. It was informed that calcium plays an important role in cell division, as well as in the growth and development of fruit trees [27]. Zhang *et al*. [79] found that calcium not only promotes plant growth, but it also increases plants' ability to resist diseases and enhances fruit flavor.

In our study, calcium nitrate was applied in combination with boric acid and showed no superiority to other treatments in terms of nematode control or productive grapevine performance. Some previous reviews indicate that the application of multiple elements (calcium, magnesium, iron, manganese, copper, and boron) through fertilizers can increase grape yields and improve fruit properties [80]. Further research using different calcium concentrations and chemical forms is required to shed light on this topic.

Many studies have documented the benefits of adding *B*. *megaterium* as arhizosphere-colonizing bacteria owing to its nematicidal activity against several plant parasitic nematodes and proposed its use as a biological control agent in certain crops [23, 36, 81, 82]. In this sense, Khalil *et al*. [10] found that the final nematode population of *Meloidogyne javanica* and the productivity of the seedless grapevine cultivar “Flame” were significantly affected by the application of the bioproduct Bio-arc^TM^ under field conditions. Several mechanisms were proposed to explain the biological control exerted by *B*. *megaterium*. For instance, Neipp and Becker [81] and Oliveira *et al*. [83] reported that *B*. *megaterium* produces metabolites that probably interfere with nematodes’ life cycle. Likewise, Huang *et al*. [84] reported the release from this microorganism of volatile nematicidal compounds that tend to reduce *M*. *incognita* infection. Other reports, such as those of Padgham and Sikora [82], reveal modifications in plant’s exudates caused by the antagonistic bacteria.

According to our results, the application of the bacterium *B*. *megaterium* through the bioproduct Bio-arc^TM^ not only contributes to nematodes biocontrol in grapevines but also provides better nutritional status. These findings are in agreement with those reported for rice [85], and sugar beet [13]. Other explanations for the mode of action of this bacterium was previously reported by Radwan [86] and López-Bucio *et al*. [87], who suggested these bacteria may have the ability to dissolve insoluble phosphorus-containing compounds in soil, enhancing P available levels and thus effectively promoting plant growth, and the induction of auxin and ethylene formation by plants, which may impact on the root system.

## 5. Conclusions

Root-knot nematodes cause significant yield losses in many grape-growing regions, and also affect fruit quality. Chemical nematicides play a significant role in minimizing these losses, but they are usually expensive and may have unwarranted effects on human health and cause environmental pollution. Safer alternative control methods to manage this pest should be developed as fast as possible, and this study contributes to that aim. We demonstrated that the application of boric acid alone was as effective as a commercial nematicide in reducing *M*. *incognita* burden in the root-soil interface of Thompson seedless grapevines, improving grape yield and fruit quality. Boric acid combined with calcium nitrate or chitosan as well the bioagent *B*. *megaterium* also decreased nematode population and enhanced grape yield and quality. Our findings may be useful for the development of more sustainable strategies to cope with nematodes infections in grapevines.

## Supporting information

S1 Data Nematode & grapevine. (XLSX)
